# Cell membrane-targeting NIR fluorescent probes with large Stokes shifts for ultralong-term transplanted neural stem cell tracking

**DOI:** 10.3389/fbioe.2023.1139668

**Published:** 2023-02-09

**Authors:** Jing Chen, Dan Li, Hongfu Li, Kongkai Zhu, Leilei Shi, Xuemei Fu

**Affiliations:** ^1^ The Eighth Affiliated Hospital, Sun Yat-sen University, Shenzhen, China; ^2^ Advanced Medical Research Institute, Shandong University, Jinan, China

**Keywords:** NIR fluorescent probe, cell membrane-targeting, large Stokes shift, ultra-strong photostability, ultralong-term tracing

## Abstract

There is an emerging therapeutic strategy to transplant stem cells into diseased host tissue for various neurodegenerative diseases, owing to their self-renewal ability and pluripotency. However, the traceability of long-term transplanted cells limits the further understanding of the mechanism of the therapy. Herein, we designed and synthesized a quinoxalinone scaffold-based near-infrared (NIR) fluorescent probe named QSN, which exhibits ultra-strong photostability, large Stokes shift, and cell membrane-targeting capacity. It could be found that QSN-labeled human embryonic stem cells showed strong fluorescent emission and photostability both *in vitro* and *in vivo*. Additionally, QSN would not impair the pluripotency of embryonic stem cells, indicating that QSN did not perform cytotoxicity. Moreover, it is worth mentioning that QSN-labeled human neural stem cells held cellular retention for at least 6 weeks in the mouse brain striatum post transplantation. All these findings highlight the potential application of QSN for ultralong-term transplanted cell tracking.

## Introduction

Human stem cells are excellent sources of cell therapy due to their self-renewal ability and pluripotency (Cossu et al., 2018; Yamanaka, 2020). Therefore, the transplantation of human neural stem cells (hNSCs) brought hope for the treatment of central nervous system (CNS) diseases such as spinal cord injury, ischemic stroke, and amyotrophic lateral sclerosis (Curtis et al., 2018; Yang H. et al., 2022; Baloh et al., 2022). The ways in which hNSCs exert their therapeutic effects are mainly through proliferation, differentiation, homing, etc. (Koch et al., 2009; Zheng et al., 2017; Hayashi et al., 2020; Pluchino et al., 2020). However, due to the lengthy self-repair process of hNSCs, the detailed therapeutic mechanism of hNSCs is still unclear. It was thought that long-term and real-time tracking of transplanted hNSCs is the first step to studying the mechanism of stem cell therapy (Zhang et al., 2016; Ceto et al., 2020). Hence, developing effective tracking tools for long-term and real-time monitoring of the differentiation process of transplanted hNSCs could help us learn more about the therapeutic mechanism of stem cells.

Currently, since optical imaging could perform high space-time resolution, fluorescent probe-based bioimaging has become the most popular real-time biological process imaging technique, which provides varied information for biological research (Li et al., 2013; Jeong et al., 2018; Zhao et al., 2018; Yang et al., 2020; Fang et al., 2021; Ghanim et al., 2021). Recently, there are some reports about smart nanomaterials which are applied in biosensing and therapy (Yi et al., 2022; Zheng et al., 2022; Zhu et al., 2022). Conventional fluorescent probes in the visible region always suffer from tissue scattering, autofluorescence, etc., resulting in low tissue penetration depth and spatial resolution. Compared with those probes in the visible region, the near-infrared (NIR) window (wavelength > 700 nm) could provide deeper tissue optical imaging, which could overcome these aforementioned issues (Hong et al., 2017; Chen et al., 2018; Huang et al., 2020; Lu et al., 2020). However, the weak photostability and resistance to photobleaching of small-molecule NIR fluorescent probes limit their long-term tracking ability *in vivo* (Zhang et al., 2012; Gong et al., 2016; Yang and Chen, 2020; Borlan et al., 2021). Therefore, developing near-infrared fluorescent probes with strong fluorescence emission intensity, good photostability, and resistance to photobleaching would be a potential method for the long-term tracking of stem cells *in vivo*.

**SCHEME 1:**
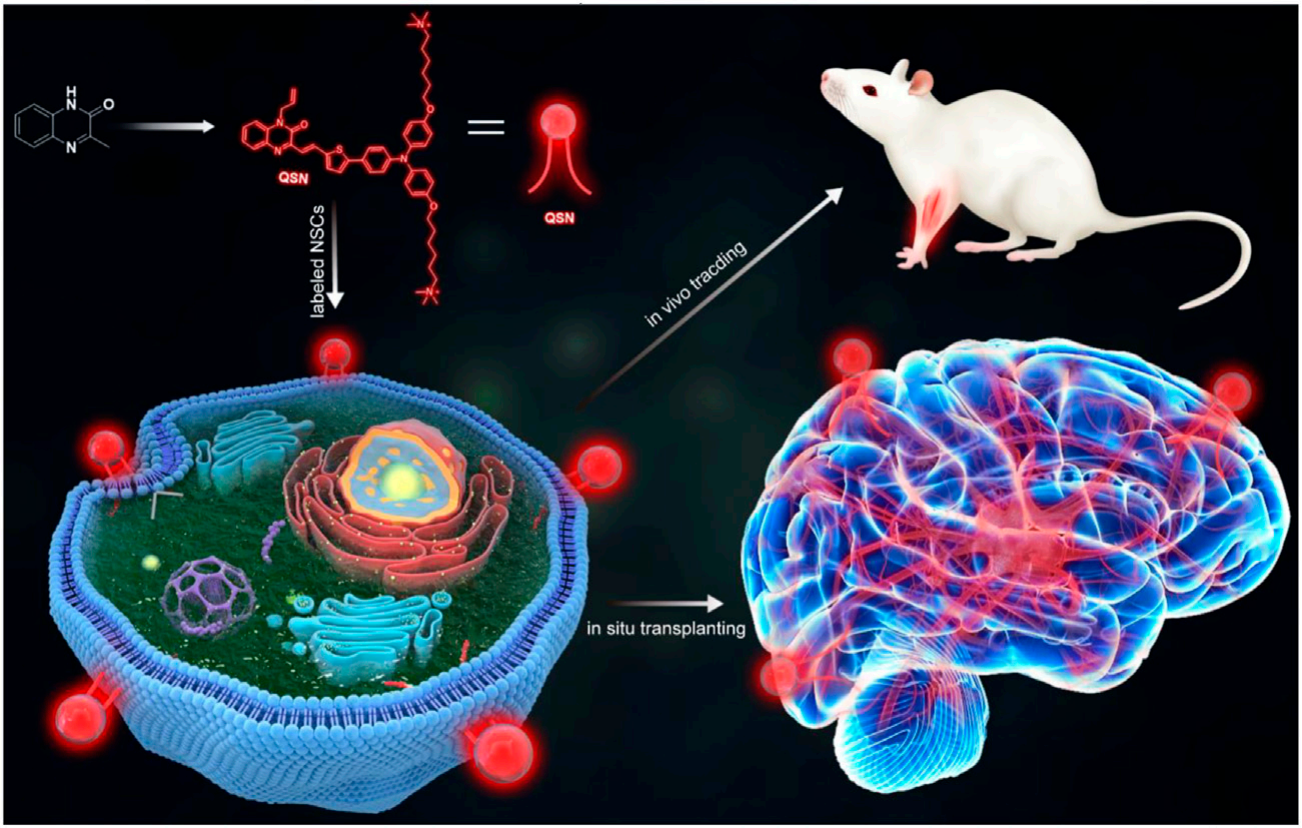
Scheme of the design and synthesis of QSN. QSN could be located in the neural stem cell membrane. Then, this membrane-targeting probe could be used for tracking the transplanted neural stem cells and ultralong-term *in vivo* imaging.

The critical issue in designing an ideal NIR probe for long-term *in vivo* tracking of stem cells was the screening for a suitable chromophore scaffold. The quinoxalinone (or quinoxalin-2-one) core, characterized by pyrazin-2(1H)-one fused to the benzene ring at two adjacent carbon atoms, could exhibit strong blue light emission and photostability. Based on this scaffold, we have successfully designed a series of probes for autophagy detection, ferroptosis identification, and Parkinson’s disease diagnosis (Shi et al., 2018; Zhang et al., 2020; Yang J. et al., 2022). Based on our previous work, a stem cell membrane-targeting NIR fluorescent probe with strong fluorescence emission, good photostability, and photobleaching resistance was designed for real-time and long-term tracking of stem cell differentiation *in vivo* (Scheme 1). In contrast to traditional fluorescent proteins, fluorescent probes could avoid the side effects of gene editing tools and could not induce genomic perturbations in stem cells (Tennstaedt et al., 2015; Anzalone et al., 2020; Alvarez et al., 2022).

## Results and discussion

### Design of QSN

First, the high-performance fluorescent probe QSN was designed and synthesized using a quinoxalinone scaffold as the core chromophore (Supplementary Scheme S1). To obtain red-to-NIR emission, good electron donors including thiophene and triphenylamine were conjugated to the quinoxalinone scaffold by which the emission of a conjugated molecular probe is expected to be greatly bathchromically shifted to the NIR region. In detail, thiophene was first covalently linked to the quinoxalinone scaffold by aldol condensation and then conjugated to triphenylamine through Suzuki–Miyaura coupling. To achieve membrane-targeting capacity, two cationic groups were further decorated on the quinoxalinone scaffold to yield QSN. The final product, QSN, was obtained with a yield of 29%, which was characterized by 1H-NMR, 13C-NMR, and mass spectra (Supplementary Figures S1, S2).

### Chemistry and photophysical properties

**FIGURE 1:**
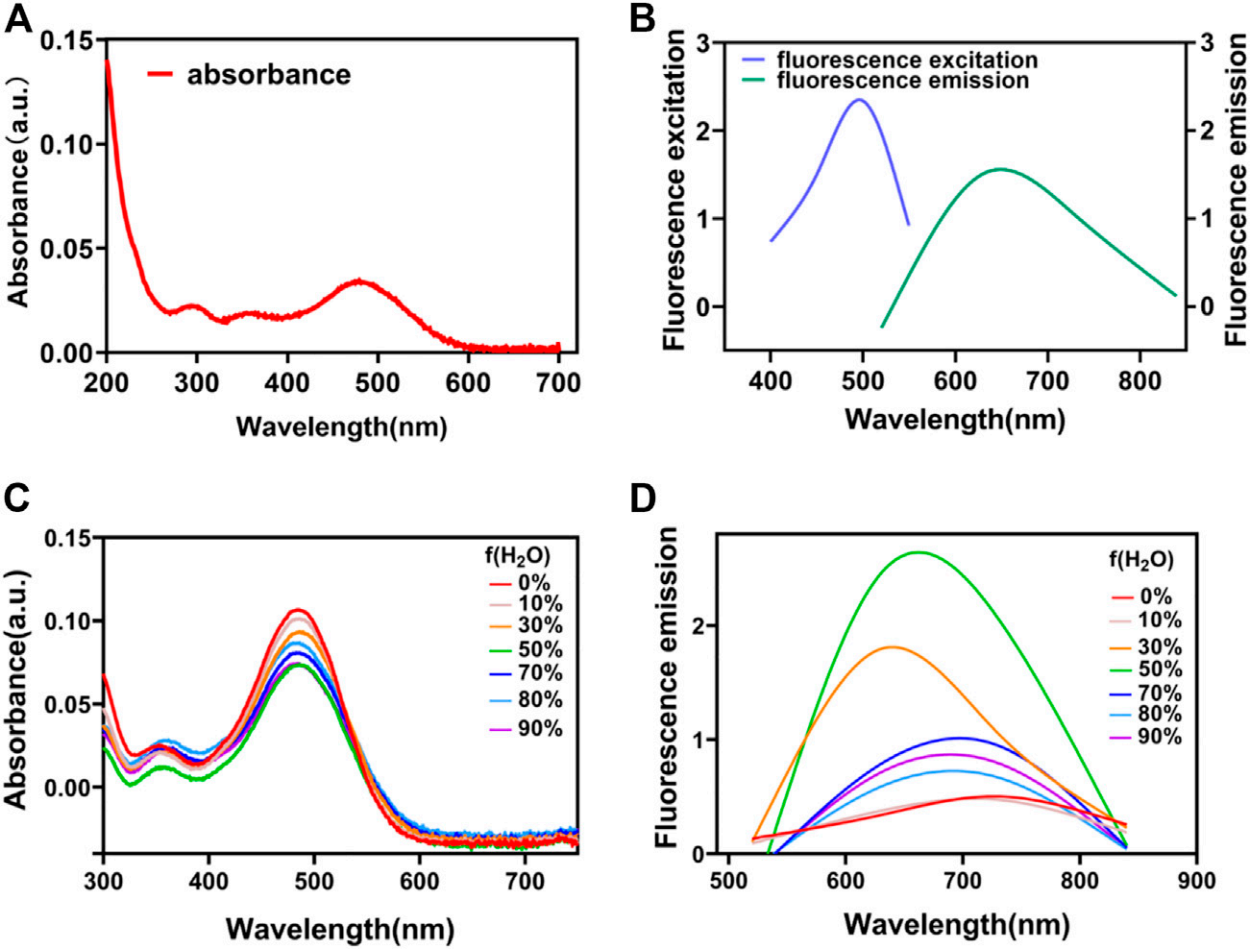
UV–vis absorption **(A)** and fluorescence spectra **(B)** of QSN. UV–vis absorption **(C)** and fluorescence spectra **(D)** of QSN in the mixed solvent with various DMSO/H_2_O ratios.

After synthesis, the optical properties of QSN were characterized. The absorption and emission spectra of QSN in dimethyl sulfoxide/water (DMSO/H2O) = 1/99 (v/v) were measured with peaks centered at 490 and 670 nm, respectively (Figures 1A, B). The large Stokes shift (typically over 80 nm) of QSN can minimize the cross talk between the excitation source and fluorescence emission, providing us with a high signal-to-noise ratio during bioimaging. In contrast to conventional fluorescent dyes with fluorescence of aggregation caused by quenching features, aggregation-induced emission (AIE) fluorophores exhibit bright fluorescence in the aggregated state but very weak fluorescence in a good solvent, making them an ideal “turn-on” fluorescent probe for bioanalysis. Herein, the AIE property of QSN was measured with different ratios of DMSO/H_2_O mixtures. When the water content was increased, both the absorption and emission intensity of QSN were greatly increased upon aggregation formation (Figures 1C, D). Notably, the emission wavelength of QSN performed a significantly bathochromic shift from 670 to 700 nm with the increase in the water ratio (Figure 1D). In addition, the simulation result indicated that the distribution of LUMO was mainly located on the terminal triphenylamine, whereas the electron densities of LUMO and HOMO were separated, resulting in a low ΔEst value (0.1022 eV) (Supplementary Figure S3). All these phenomena indicated that QSN exhibited in an AIE-active manner with strong NIR emission in an aqueous solution, indicating that QSN could be a potential tracking tool for monitoring biological processes.

### Cell viability study

With the satisfactory photophysical property of QSN in hand, the potential cytotoxicity of QSN was first evaluated using cell counting kit-8 (CCK-8). The human embryonic cell line H7 was treated with QSN at various concentrations and incubated for 24 h. It could be found that H7 viability still remained at a high value (≈89%) even after incubating with QSN at a concentration of up to 100 μM for 24 h, which means that QSN did not exhibit obvious cytotoxicity (Supplementary Figure S4A). In addition, QSN also exhibited a low cytotoxicity capacity for H7-derived NSCs (H7NSCs). These results showed that H7NSCs could maintain high cell viability up to 90% even after 15 min of incubation with 300 μM QSN (Supplementary Figure S4B). All these outcomes brought a good signal for the further biological research and application of QSN, owing to its high biocompatibility.

### Cellular uptake and confocal imaging study

**FIGURE 2:**
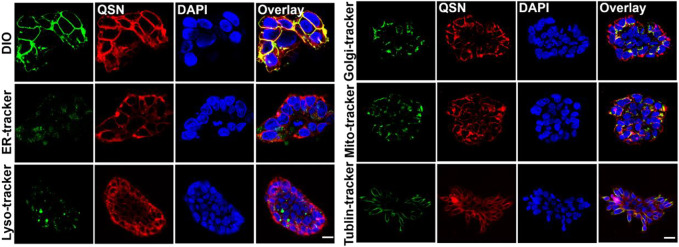
Confocal imaging of human embryonic stem cells-7 (H7) co-stained with QSN (50 μM), DiO, ER-Tracker, lysosome tracker, Golgi-Tracker, MitoTracker, and Tublin Tracker. Cells were incubated with QSN for 15 min, and different organelle trackers were added and incubated for an additional 30 min to 1 h. DIO, ER-Tracker, and MitoTracker were incubated with cells for 30 min. Lysosome tracker, Golgi-Tracker, and Tublin Tracker were incubated with cells for 1 h according to the protocols. Scale bar: 20 µm. All experiments were carried out three times independently.

Then, in order to identify where QSN binds to the cells, confocal imaging-based colocalization experiments were conducted by co-staining the cells with both QSN and other organelle trackers. First, the localization of QSN in cells was investigated by co-staining with DiO, a cell membrane fluorescent probe. H7 cells were incubated with QSN (50 μM) for 15 min, followed by DiO (1:1000) for another 30 min. The red fluorescence of QSN almost completely targeted that of the DiO (green), revealing the excellent cell membrane-targeting capacity of QSN. In addition, H7 cells were incubated with QSN and other different organelle trackers such as Mito and Tublin, providing fluorescent staining of ER, lysosome, and Golgi apparatus, which were added and incubated for further 30 min or 1 h. On the contrary, QSN could barely target the other organelle trackers except the plasma membrane, indicating that QSN has a highly specific cytomembrane-dyeing ability (Figure 2). Additionally, QSN showed the same cytomembrane-staining capacity for H7NSCs as well (Supplementary Figure S5B). These results corroborated our hypothesis that QSN could perform as an excellent cell membrane-targeting fluorescent probe for bioimaging.

### *In vitro* photostability study

**FIGURE 3:**
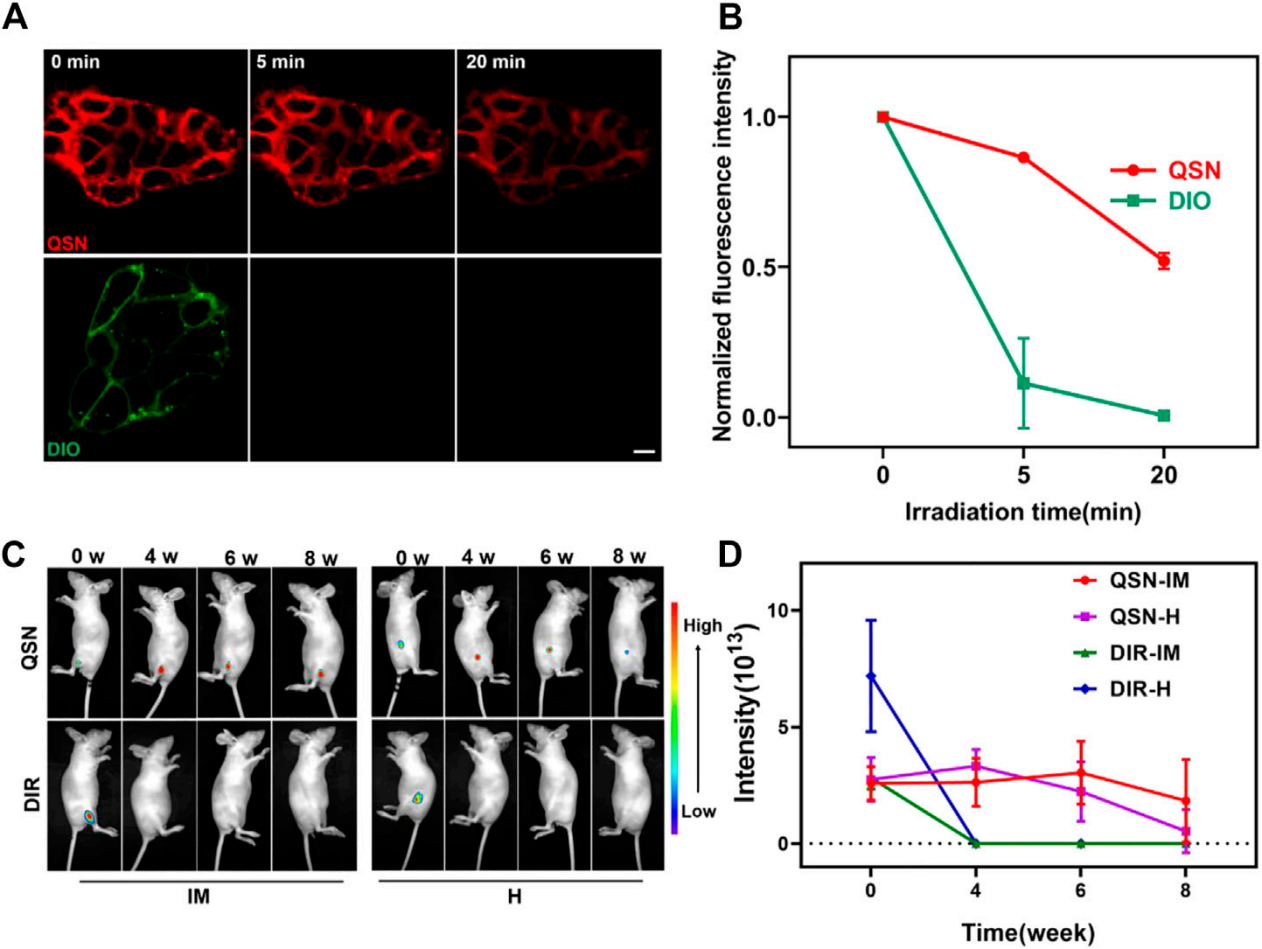
**(A,B)** Comparison of photostability of QSN (upper) and DiO (below) when incubating the samples with H7 and exposing to laser irradiation for 20 min, respectively. Scale bar: 10 µm. **(C,D)** Long-term *in vivo* imaging of time-dependent whole-body imaging of H7NSC-bearing nude mice (*n* = 3) after treatment with QSN (300 μM) and DiR (1:2500) *via* intramuscular (IM) injection and hypodermic (H) injection.

Hence, for further confirming the potential of QSN to be applied as a stable imaging agent, a study on photostability of QSN was conducted by comparing the cytomembrane staining of H7 using QSN (upper) and commercially available DiO (bottom) *via* persistent laser irradiation for 20 min. The fluorescence intensity of DiO swiftly decreased and became inconsiderable after 5 min of irradiation because of photobleaching (Figures 3A, B). For the QSN probe, strong fluorescence signals could be observed with no distinct signal decay even after 20 min of irradiation. This result demonstrated that QSN exhibited superior photobleaching resistance, indicating its great potential in bioimaging applications.

### Teratoma formation study

The pluripotency of hESCs makes them an important source in regenerative medicine. Teratoma formation assay is the gold standard for *in vivo* assessment of pluripotency of hESCs. To determine whether labeling with QSN affects the pluripotency of H7, we compared the capability of QSN-labeled H7 to form teratomas with H7-wild type (H7-WT). QSN-labeled H7 cells were implanted subcutaneously into the right flank of non-obese diabetic/ShiLtJGpt-Prkdcem26Cd52Il2rgem26Cd22/Gpt (NCG) mice. As a positive control, the H7-WT was implanted into the left flank of the same NCG mice. We found that QSN-labeled H7 could form teratomas that contain cells derived from each of the three germ layers as same as H7-WT, 6 weeks after transplantation (Supplementary Figure S6), demonstrating that labeling with QSN would not impair the pluripotency of hESCs. In other words, QSN could be used for tracing stem cells without affecting the differentiation potential of stem cells.

### *In vivo* imaging study

As mentioned previously, the therapeutic mechanisms of hNSCs transplanted for the treatment of CNS diseases remain unclear. Owing to the superior photostability and photobleaching resistance of QSN *in vitro*, we suspected that it will be suitable for long-term *in vivo* tracing, which may be conducive to studying the therapeutic mechanism of hNSC transplantation. For confirming this conjecture, first, we labeled H7NSCs with QSN. H7NSCs were incubated with QSN (300 μM) and commercially available DiR dye (1:2500) individually for 15 min at 37°C (labeled as 0 week). Then, the treated cells were transplanted into the nude mice (*n* = 3 per group) *via* intramuscular (IM) injection and hypodermic (H) injection, respectively. After that, the *in vivo* imaging system was used for monitoring fluorescence intensity every 2 weeks. As shown in Figures 3C, D and Supplementary Figure S7A, the fluorescence signal of subcutaneous QSN could still be detected 10 weeks later. Amazingly, the signal is retained in the muscle for even up to 12 weeks. However, the commercially available DiR-dyed fluorescence signals became negligible at the second week and vanished after 4 weeks. In summary, compared with the commercial dye, QSN also showed a longer tracing capability *in vivo*. This capability makes ultralong-term *in vivo* tracing possible.

### *In situ* transplantation study

**FIGURE 4:**
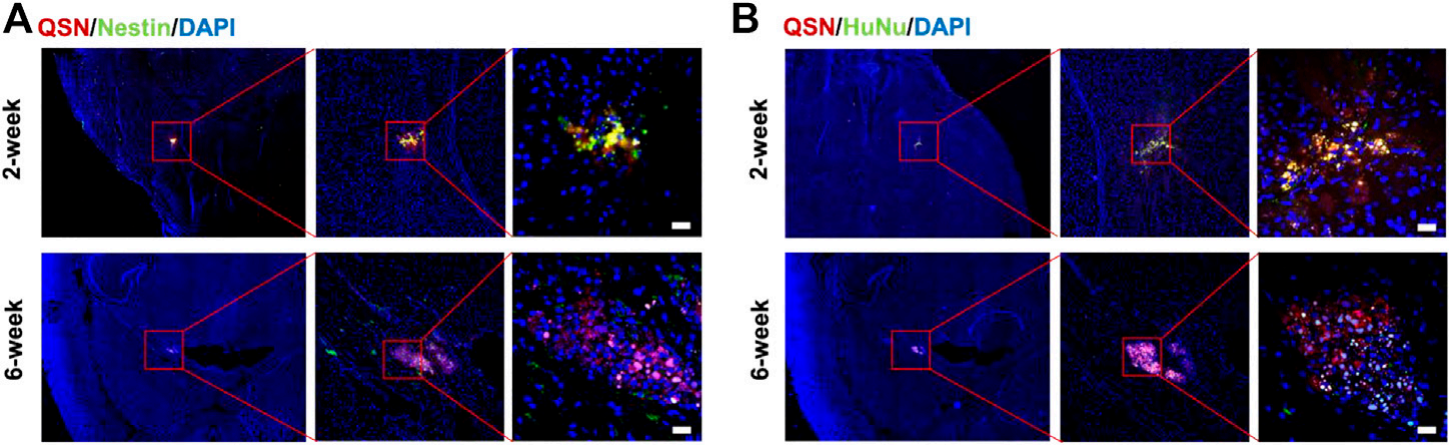
Transplantation of QSN-labeled H7NSCs in the mouse brain. QSN-labeled H7NSCs in the mouse brain at 2 and 6 weeks post transplantation were co-stained with **(A)** Nestin, a typical marker of human neuronal stem cells, **(B)** human nuclear marker, and DAPI. Scale bar: 20 μm.

Considering that the transplantation of hNSCs is usually administered *in situ*, we next investigated the tracing capability of QSN in the brain. At first, we labeled H7NSCs with QSN and DiR, respectively, as previously described. Subsequently, the labeled H7NSCs were transplanted into the null mice brain striatum. At 2 weeks and 6 weeks post transplantation, mice were sacrificed for immunostaining analysis. At respective time points, mice transplanted with DiR-labeled H7NSCs were used as controls. After 2 weeks, grafts were observed with the strong QSN signal when co-stained with the human-specific Nestin antibody and human nuclear antibody, respectively, while fluorescent signals of DiR were almost undetectable (Figure 4; Supplementary Figure S8). Surprisingly, the fluorescence emitted by QSN can even be detected at 6 weeks (Figure 4). This is consistent with our previous results obtained by the *in vivo* imaging system, certifying that QSN exhibited outstanding photostability and endurance to photobleaching compared to commercially available dyes not only *in vitro* but also *in vivo*, suggesting the considerable scope of QSN for *in vivo* ultralong-term tracking of biological processes.

### *In vivo* biochemical analysis study

To determine whether QSN could be a potential probe for further clinical development, biosafety would be a key issue. First, it could be noticed that the body weight of mice did not change significantly after the administration of QSN (Supplementary Figure S7B). Next, we compared H&E staining of organs with QSN-labeled H7NSCs transplanted into null mice with that of normal null mice. We found that QSN-labeled H7NSCs posed nearly no impact on the organs (Supplementary Figure S9). In addition, we performed blood biochemistry analysis to evaluate the liver and kidney function of mice. Among the three groups, there was no significant difference in the levels of alanine transaminase (ALT), aspartate transaminase (AST), albumin (ALB), serum creatinine (Scr), and blood urea nitrogen (BUN) (Supplementary Figure S10). These results indicate that QSN-labeled H7NSCs are biocompatible *in vivo*, thus representing a safe fluorescent probe for *in vivo* application.

## Conclusion

In summary, we developed a quinoxalinone-based cell membrane-targeting fluorescent probe with an AIE-active property, strong NIR emission, high photostability, and large Stokes shifts. Additionally, QSN exhibited negligible cellular and systematic toxicity both *in vitro* and *in vivo*. Furthermore, QSN could efficiently label the stem cells with strong fluorescence emission and photobleaching resistance. Given these excellent characteristics, we trust that QSN could be applied to track biological processes in a long-term fashion. Indeed, it was verified that QSN-labeled stem cells held cellular retention for at least 6 weeks in the mouse brain striatum post transplantation. All these findings demonstrated that QSN would be a potential tool for providing us with a methodology to track the *in vivo* biological process and helping us study the mechanism of *in vivo* stem cell therapy.

## Data Availability

The original contributions presented in the study are included in the article/Supplementary Material; further inquiries can be directed to the corresponding authors.
