# Supplementary material for: Cell membrane-targeting NIR fluorescent probes with large Stokes shifts for ultralong-term transplanted neural stem cell tracking
Source: Front Bioeng Biotechnol. 2023 Feb 9;11:1139668. doi: 10.3389/fbioe.2023.1139668 (PMC9948019; doi:10.3389/fbioe.2023.1139668)
Supplement: Supplementary file 1 [file DataSheet1.docx]

Supporting information

Cell Membrane Targeting NIR Fluorescent Probe with Large Stokes Shift for Ultralong-Term Transplanted Neural Stem Cell Tracking

**Jing Chen^1†^, Dan Li ^1†^, Hongfu Li^1^, Kongkai Zhu^2*^, Leilei Shi^1*^ and Xuemei Fu^1*^**

*** Correspondence:**

Kongkai Zhu email:[hkhhh.k@163.com](mailto:hkhhh.k@163.com)

Leilei Shi email:[shillei@mail.sysu.edu.cn](mailto:shillei@mail.sysu.edu.cn)

Xuemei Fu email:fxmzj2004@163.com

**Experimental Section:**

**Materials:**Benzene-1,2-diamine, ethyl-2-oxopropanoate and 3-bromoprop-1-ene were purchased from Adamas (China), and 5-bromothiophene-2-carbaldehyde, 4,4'-((4-bromophenyl) azanediyl) diphenol, 1,6-dibromohexane, trimethylamine and Tetrakis(triphenylphosphine) palladium were purchased from J&K chemical. All reagents were of analytical grade and used without purification. Cell-membrane tracker, Endoplasmic reticulum (ER)-tracker, Lyso-Tracker Green, Mito-tracker, and Hoechst were purchased from Thermo Fisher, and Cell Counting Kit-8 (CCK8) were purchased from Sigma.

**Instruments:**^1^H and 13C nuclear magnetic resonance spectroscopy (1H and 13C NMR) was employed using a Mercury plus 400 MHz spectrometer (Varian, USA) with dimethyl sulfoxide-d6 (DMSO-d6) and chloroform-d6 (CDCl3) as solvents. Liquid chromatography-mass spectrometry (LC-MS) was performed on a Water ACQUITY UPLC system equipped with a binary solvent delivery manager and a sample manager coupled to a Waters Q-TOF Premier Mass Spectrometer equipped with an electrospray interface (Waters Corporation, Milford, MA). Milli-Q water was supplied by Milli-Q Advantage A10 (Millipore Corporation, France). UV-vis absorption spectrum was recorded on UV-1700 (Shimadzu, UV-1700, Japan). The fluorescence spectrum was analyzed using a multifunctional microplate reader (Thermo Scientific Varioskan LUX, USA). The fluorescent signals were observed and collected by confocal laser scanning microscopy (Zeiss LSM880, Germany). The in vivo fluorescent imaging was performed using an IVIS (AniView100, China).

**Synthesis of compound 1: 3-methylquinoxalin-2(1H)-one (1a)1.**The *o*-Phenylenediamine (0.1 mol, 10.8 g) was suspended in anhydrous ethanol (150 mL) and the mixture was cooled in an ice bath. A solution of ethyl pyruvate (0.12 mol, 13.92 g) in anhydrous ethanol (10 mL) was added dropwise over a period of 20 min under stirring. The resulting solution was allowed to react at room temperature for 12 h. The resulting precipitate was filtered, washed with ethanol, and dried under vacuum to give the product **1** (13.6 g, yield: 85%) as a white solid which was pure without purification. ESI-MS m/z:161.6 (M+).

**Synthesis of compound 2: 1-allyl-3-methylquinoxalin-2 (1H)-one (2a).**Compound 1a (20 mmol, 3.2 g) and K2CO3 (24 mmol, 3.31 g) were suspended in acetone (100 mL). Then, a solution of 3-bromoprop-1-ene (24 mmol, 2.88 g) was added dropwise into the mixture, which was stirred in an oil bath at 62°C for 12 h. After completion of the reaction, the solvent was removed via rotary evaporator and the residue was partitioned between distilled water (20 mL) and ethyl acetate (EA) (40 mL). The organic layer was separated and dried over anhydrous MgSO4, filtered, and concentrated via rotary evaporator to obtain a crude product that was further purified via silica gel chromatography (hexane: EA = 20:1, v:v) to afford 2.21 g of 2a as a white solid, yield 55%. 1H NMR (400 MHz, CDCl3, δ): 7.83 (dd, J1 = 8.0 Hz, J2 = 2.0 Hz, 1H), 7.46-7.50 (m, 1H), 7.32-7.33 (m, 1H), 7.28-7.29 (m, 1H), 5.88-5.98 (m, 1H), 5.24-5.27 (m, 1H), 5.13-5.18 (m, 1H), 4.90 (d, J = 8.0 Hz, 2H), 2.61 (s, 3H) ppm; HRMS: m/z (ESI) calculated for C12H12N2O (M+), 201.0950, found 201.0984.

**Synthesis of compound 3: E- 1-allyl-3-(2-(5’-bromo-[2-2’-bithiophen]-5-yl)vinyl)quinoxalin-2 (1H)-one (3a).**A solution of compound 2a (2.5 mmol, 500 mg) in acetic acid (10 mL) was supplemented with 5-bromothiophene-2-carbaldehyde (3 mmol, 570 mg), and catalytic concentrated sulfuric acid. The resulting solution was heated to 50 °C and reacted for 8 h. The reaction mixture was concentrated using a rotary evaporator, and water (20 mL) and ethyl acetate (50 mL) were added. The organic layer was collected, and the water phase was basified with K2CO3 and extracted with ethyl acetate, and the combined organic phase was dried via anhydrous MgSO4, and purified by silica chromatography (PE: EA = from 20:1 to 10:1) to yield 522 mg of 3a as a yellow solid, yield 56%. 1H NMR (400 MHz, CDCl3, δ): 8.08-8.12 (m, 1H), 7.78 (d, J = 8.0 Hz, 1H), 7.52-7.57 (m, 2H), 7.42-7.45 (m, 1H), 7.31-7.37 (m, 3H), 7.24-7.27 (m, 2H), 5.87-5.98 (m, 1H), 5.18 (dd, J1 = 12.0 Hz, J2 = 4.0 Hz, 1H), 5.09 (dd, J1 = 12.0 Hz, J2 = 4.0 Hz, 1H), 4.86-4.90 (m, 2H) ppm; HRMS: m/z (ESI) calculated for C17H13BrN2OS (M+), 373.2669, found 373.0002.

**Synthesis of compound 4: 4-((6-bromohexyl)oxy)-N-(4-((6-bromohexyl)oxy)phenyl)-N-(4-(4,4,5,5-tetramethyl-1,3,2-dioxaborolan-2-yl)phenyl)aniline.** Preparation of compound 4 was from starting raw materials 4-bromo-N,N-bis(4-methoxyphenyl)aniline. After demethylation, substitution reaction and Suzuki coupling, we can obtain compound 4 as a brown solid, yield 61%. ^1^H-NMR (400 MHz, d^6^-DMSO) δ 7.48 (d, J = 7.8 Hz, 2H), 7.34 (d, J = 8.0 Hz, 4H), 7.21 (d, J = 7.8 Hz, 2H), 6.91 (d, J = 8.0 Hz, 4H), 3.98-3.92 (m, 4H), 3.91 (d, J=16.0 Hz, 4H), 3.45 (d, J=16.0 Hz, 4H), 1.83-1.80 (m, 4H), 1.74-1.69 (m, 4H), 1.43-1.38 (m, 4H), 1.24-1.19 (m, 4H), 1.23 (s, 12H) ppm. High resolution Mass Spectrum (in Methanol) (C_20_H_18_BrNO_2_): m/z found 385.1524.

**Synthesis of compound 5: (E)-1-allyl-3-(2-(5-(4-(bis(4-((6-bromohexyl)oxy)phenyl)amino)phenyl)thiophen-2-yl)vinyl)quinoxalin-2(1H)-one.**A mixture of compound 3 (1 mmol, 374 mg), K2CO3 (4 mmol, 5.52 g) was supplemented with compound 4 (1.2 mmol, 872 mg), tetrakis(triphenylphosphine)palladium) (0.1 mmol, 114 mg), distilled water (15 mL) and 1,4-dioxane (30 mL). The reaction mixture was stirred at 80°C under argon atmosphere for 72 h. The reaction mixture was concentrated using a rotary evaporator, and water (20 mL) and ethyl acetate (30 mL) were added; the organic layer was collected and dried with anhydrous MgSO4 and purified using silica chromatography (PE: EA = from 100:1 to 20:1) to yield compound 5 as a red solid, yield 43%. 1H-NMR (400 MHz, d6-DMSO) δ 7.98 (d, J = 8.0 Hz, 1H), 7.61 (d, J = 8.0 Hz, 1H), 7.49-7.42 (m, 5H), 7.37-7.33 (m, 2H), 7.22-7.18 (m, 1H), 7.13-7.09 (m, 1H), 7.06-7.03 (m, 3H), 6.90-6.85 (m, 2H), 6.83-6.79 (m, 3H), 6.02-5.96 (m, 1H), 5.28 (d, J=8.0, 1H), 5.18 (d, J=14 Hz, 1H) 4.95 (s, 1H), 4.75 (s, 1H), 3.56-3.50 (m, 4H), 1.62-1.54 (m, 6H), 1.49-1.46 (m, 2H), 1.45-1.28 (m, 4H), 1.29-1.22 (m, 2H), 1.24-1.19 (m, 4H) ppm.

**Synthesis of final compound QSN: (E)-6,6'-((((4-(5-(2-(4-allyl-3-oxo-3,4-dihydroquinoxalin-2-yl)vinyl)thiophen-2-yl)phenyl)azanediyl)bis(4,1-phenylene))bis(oxy))bis(N,N,N-trimethylhexan-1-aminium) chlorid.**A solution of compound 5 (0.2 mmol, 179 mg) in acetonitrile (10 mL) was supplemented with sodium bicarbonate (2 mmol, 168 mg) and Trimethylamine hydrochloride (0.5 mmol, 47.5 mg). The resulting solution was reflux for 24 h. The reaction mixture was concentrated using a rotary evaporator, then the residue was washed by water (20 mL) and diethyl ether (20 mL) for 3 times under ultrasound to yield 161 mg of QSN as a deep red solid, yield 87%. 1H-NMR (400 MHz, d6-DMSO) δ 8.26(d, J = 8.4 Hz, 1H), 7.84 (d, J = 7.8 Hz, 1H), 7.47-7.40 (m, 5H), 7.35-7.31 (m, 2H), 7.22 (m, 1H), 7.13 (m, 1H), 7.08-7.03 (m, 3H), 6.91-6.83 (m, 2H), 6.84-6.80 (m, 3H), 6.00-5.92 (m, 1H), 5.27 (d, J=8.4, 1H), 5.18 (d, J = 14.0 Hz, 1H) 4.94 (s, 1H), 4.74 (s, 1H), 3.98-3.92 (m, 4H), 3.89 (s, 12H), 3.45 (s, 4H), 3.43 (s, 6H), 1.54-1.47 (m, 6H), 1.45-1.43 (m, 2H), 1.44-1.40 (m, 2H), 1.33 (s, 2H), 1.30-1.26 (m, 4H) ppm. 13C-NMR (400 MHz, d6-DMSO): δ = 160.4, 158.2, 155.6, 154.9, 145.9, 140.1, 131.9, 131.4, 131.3, 130.3, 129.8, 129.3, 129.1, 128.8, 127.7, 126.9, 126.5, 125.8, 123.9, 122.5, 120.9, 120.6, 119.5, 118.0, 115.6, 114.2, 70.5, 68.0, 67.6, 53.4, 52.0, 50.8, 45.0, 44.0, 32.5, 31.4, 30.2, 29.7, 29.2, 27.1, 25.5, 23.1, 21.7 ppm. High resolution Mass Spectrum (in Methanol) (C53H67N5O3S): m/z found 853.3099.

**Cell culture and differentiation****.** Human embryonic stem cells-7 (H7) was obtained from ATCC and cultured with mTeSR1 medium (StemCell Technologies, Canada) on Matrigel (Corning, United States)-coated dishes at 37 ℃ with 5% CO2 humidified atmosphere.

Neural stem cells originated from H7 (named as H7NSCs) were achieved via small molecule inhibitors. Briefly, at day 0, dissociated single H7 were cultured in mTeSR1 medium supplemented with Rho-associated kinase (ROCK) inhibitor (Tocris, England) for 24 h to adhering to the wall. At day 3, H7 plated on Matrigel at about 30% confluence were treated with NSC induction medium containing 1:1 Advanced Dulbecco’s Modified Eagle’s Medium/F12 (DMEM/F12, Gibco, United States): Neurobasal (Gibco) (1:1), non-essential amino acids (Gibco), Gluta-MAX (Gibco), N2 supplement (Gibco), B27 supplement (Gibco) with 4 μM CHIR99021 (Selleck, United States), 3 μM SB431542 (Selleck), 0.1 μM Compound E (MCE, United States), 5 μg/mL BSA (Sigma-Aldrich, United States)and 10 ng/mL human LIF (Miltenyi, Germany) for 7 days. The cells were then split 1:3 for the next six passages using Accutase (Gibco) and cultured in NSC culture medium which is similar to the NSC induction medium except 3 μM CHIR99021, 2 μM SB431542 and without Compound E on Matrigel-coated plates. H7NSCs cultured about day 21 to 28 successfully identified by Nestin and Pax6, two classical markers of neural stem cells, would be use to next labeled experiment (Figure S5A).

**In vitro cytotoxicity studies****.** To determine the optimal QSN labeling condition for H7/H7NSCs, cell viability assays were performed using Cell Counting Kit-8 (CCK8). At day 0, dissociated single cells were cultured in medium supplemented with ROCK inhibitor for 24 h in 96-well plates. Then, cells were treated with QSN at various concentrations (from 0 μM to 300 μM) and incubated for 24 h/15 min. After that, 10 μL CCK8 solutions were added to the 96-well plates. The obtained solution was measured in a multifunctional microplate reader at a wavelength of 450 nm after 1h incubation at 37 °C.

**Confocal imaging of cells stained by dyes.** H7/ H7NSCs were seeded in confocal dishes and cultured for 48 h in mTeSR1 medium/NSC culture medium. QSN (50 μM/20 μM) and probes were added to each dish and cultured for various times. Cells were washed with PBS three times, and 1 mL of PBS was finally added before imaging. Cells were imaged with a 40x objective lens. The fluorescence of DIO (Thermo Fisher, United States), ER tracker (Thermo Fisher), Lysosome tracker (Thermo Fisher), Golgi tracker (Thermo Fisher), Mito tracker (Thermo Fisher), Tublin tracker (Thermo Fisher) was excited with a 488nm laser with emission collected at 493-516 nm. The fluorescence of DAPI (Thermo Fisher) was excited with a 405 nm laser with emission collected at 410-480 nm. The fluorescence of QSN was excited with a 561 nm laser with emission collected at 670-750 nm.

**Animals.** The protocol for the use of experimental animals was approved by Animal Ethics Committee of SUN YAT-SEN University (Approved number: A2020-030-01). Five weeks old female nude mice and non-obese diabetic/ShiLtJGpt-Prkdcem26Cd52Il2rgem26Cd22/Gpt (NCG) mice (GemPharmatech, Jiangsu, China) were used for cell transplantation. All mice were maintained in a pathogen-free facility and exposed to 12 h light/dark cycle with food and water. Efforts were made to minimize animal suffering and the number of animals used.

**Teratoma formation assay.** H7 were dissociated with Accutase, single cells were harvested and incubated with QSN (300 μM) for 15 min at 37 ℃, then washed with PBS. 2x106 cells labeled with/without QSN were resuspended with 100 μL Matrigel, and implanted in NCG mice subcutaneously. About 6-8 weeks later, teratoma were harvested for subsequently analysis.

**In vivo imaging study****.** H7NSCs were dissociated as single cells with Accutase, then incubated with 300 μM QSN and 1:2500 commercially available DIR dye (AAT Bioquest, United States) individually for 15 min at 37 °C. After washed with PBS, cells were resuspended in Matrigel for intramuscular transplanting (1x10^6^ cells in 100 μL Matrigel) or hypodermic transplanting (1x10^6^ cells in 100 μL Matrigel). Fluorescence intensity was monitored biweekly using an in vivo imaging system with appropriate wavelength (For QSN: λex = 530 nm, λem = 680 nm; For DIR: λex = 740 nm, λem = 820 nm).

**In situ transplantation of H7NSCs****.** For in situ transplantation, nude mice were randomly divided into normal control, QSN and DIR groups. H7NSCs were harvested, incubated with QSN or DIR as described above. The mice were anesthetized with 1% Pentobarbital (10 μL/g), and then 2x10^5^ cells in 5 μL PBS were injected slowly in the striatum with micro syringe. The injection coordinate is the right striatum (anterior 1.6 mm, lateral -2.0 mm, ventral 3.6 mm). The micro syringe was slowly removed 1 min after the termination of injection. Mice transplanted with QSN or DIR labeled H7NSCs were sacrificed at 2 weeks and 6 weeks for further analysis.

**Immunohistochemistry staining.** After being anesthetized, the mice were transcranial perfused with 4% paraformaldehyde buffer (PFA), and then the brains were removed, fixed in 4% PFA overnight, after that cryoprotected in 30% sucrose overnight at 4 ℃ follow by cryo-embedding in optimal cutting-temperature compound (OCT). Brain samples were cut into 8 μm thick sections. Brain sections were permeabilized in 0.3% Triton X-100 for 15 min at room temperature and blocked with 2% BSA for 30 min. After overnight incubation at 4 ℃ with primary antibodies mouse anti-human Nestin (1:3200, Cell Signaling Technology, United States) or mouse anti-human nuclear (1:200, Abcam, England). The sections were wash with 0.1% DPBS-Tween three times, then incubated at room temperature with the secondary antibody, anti-mouse IgG conjugated with Alexa Fluor@ 488 (1:1000, Cell Signaling Technology) for 1 h. Finally, the sections were washed with 0.1% DPBS-Tween three times again and mounted with Antifade Mounting Medium with DAPI (Vector, United States). Images were obtained using a laser scanning confocal microscope.

**Biochemical Analysis.** Blood samples were collected from the abdominal aorta, allowed to clot for 30 min at room temperature and then centrifuged at 3,000 rpm and 25 ℃ for 10 min to obtain the serum. Albumin (ALB), alanine aminotransferase (ALT), aspartate aminotransferase (AST), serum creatinine (Scr) and blood urea nitrogen (BUN) were determined using standard local laboratory techniques.

**Histological Studies.** The excised teratoma, heart, liver, spleen, lung and kidney in each group were fixed using 4% PFA, embedded in paraffin and sectioned into 5 μm thick slices. The sections were stained with H&E for histological examinations and imaged by using a digital slide scanner (Pannoramic MIDI, 3DHISTECH).

**Statistical Analysis.** The results were expressed as the mean standard deviation (SD). Error bars were reported as mean ± SD. The differences between groups were evaluated by the Graphpad Prism 9 software and analyzed with the Student t-test or one-way analysis of variance (ANOVA) where appropriate. The difference was considered as statistical significant (*p < 0.05) and very significant (**p < 0.01, ***p < 0.001). NS is indicated as no significant difference.

**Supplementary Figures**





**Scheme S1.** Design and synthesis of cell membrane targeting probe (QSN).1-allyl-3-methylquinoxalin-2 (1H)-one staring material, which yielded QSN after aldol condensation, Suzuki-Miyaura coupling and substitution reaction.


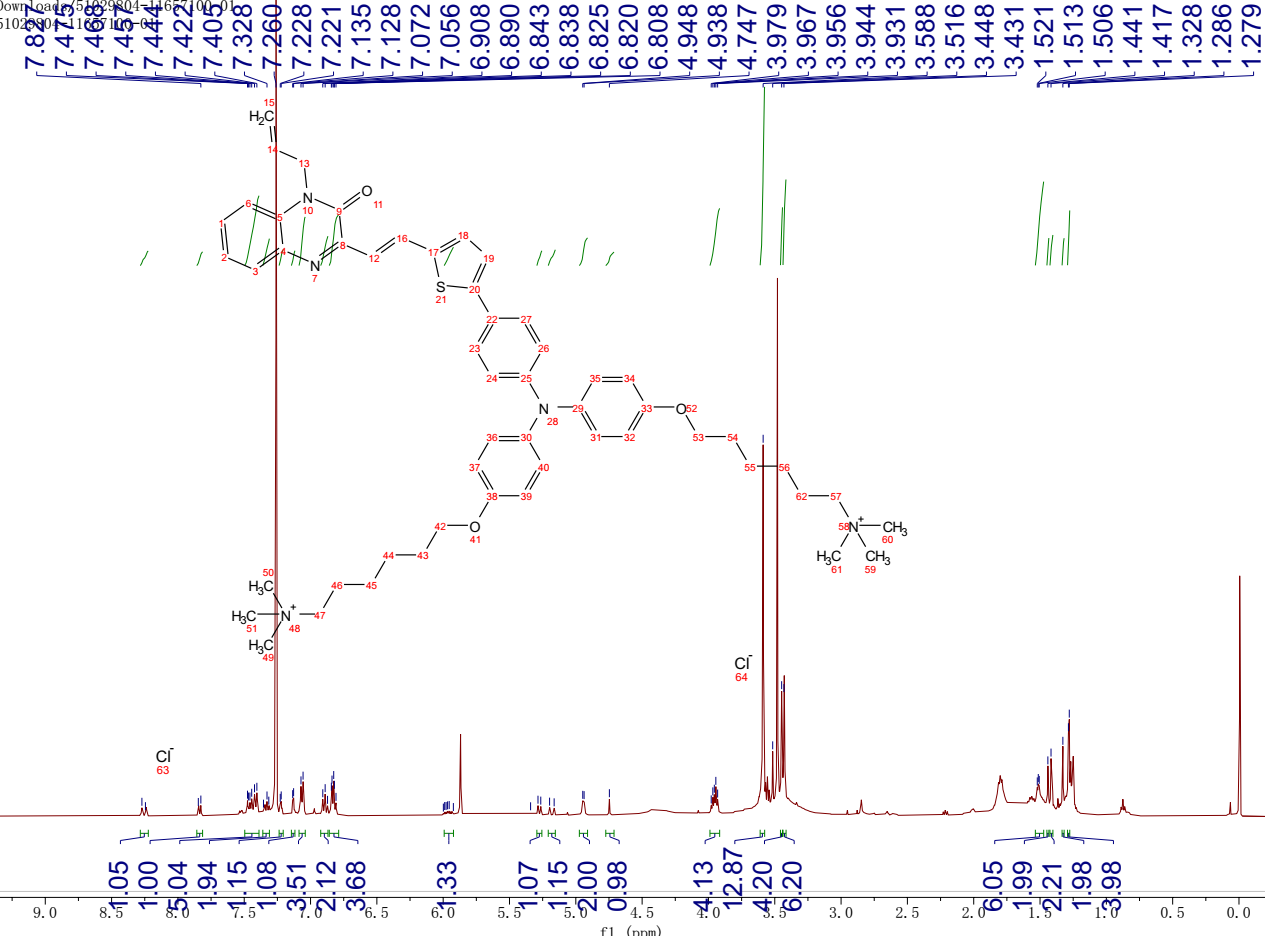


**Figure S1.** 1H NMR spectrum of QSN in Deuterated chloroform (CDCl3).


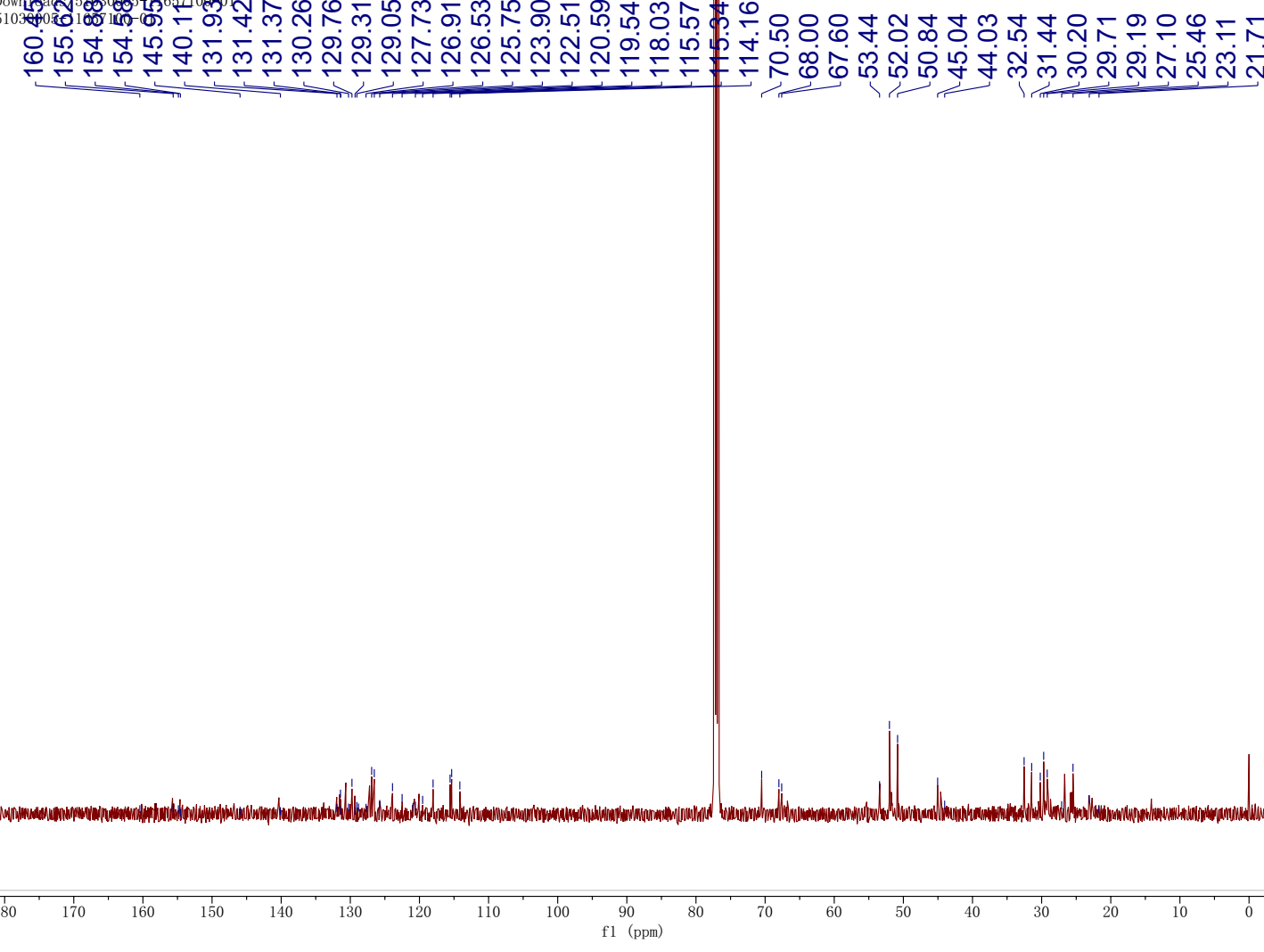


**Figure S2.** 13C NMR spectrum of QSN in Deuterated chloroform (CDCl3).

**
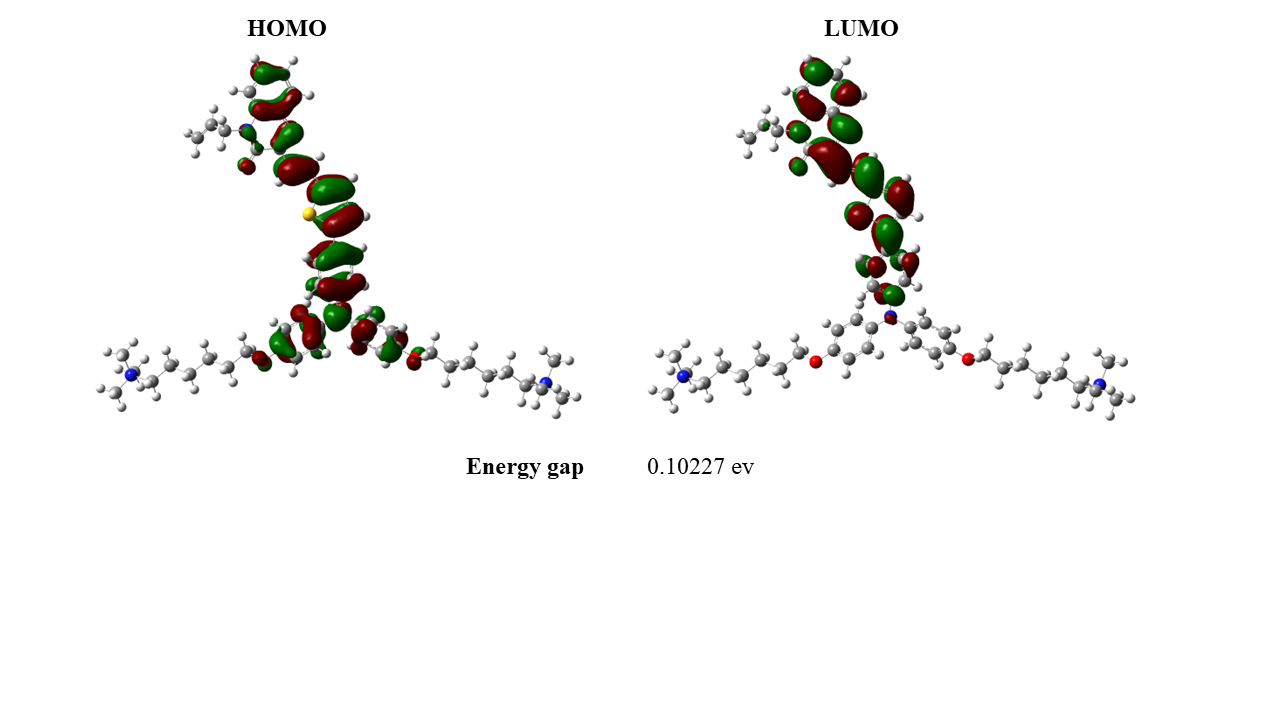
**

**Figure S3.** HOMO−LUMO distributions of QSN. The optimized chemical structures for the HOMO and LUMO were calculated at the S1 state by density functional theory (DFT).


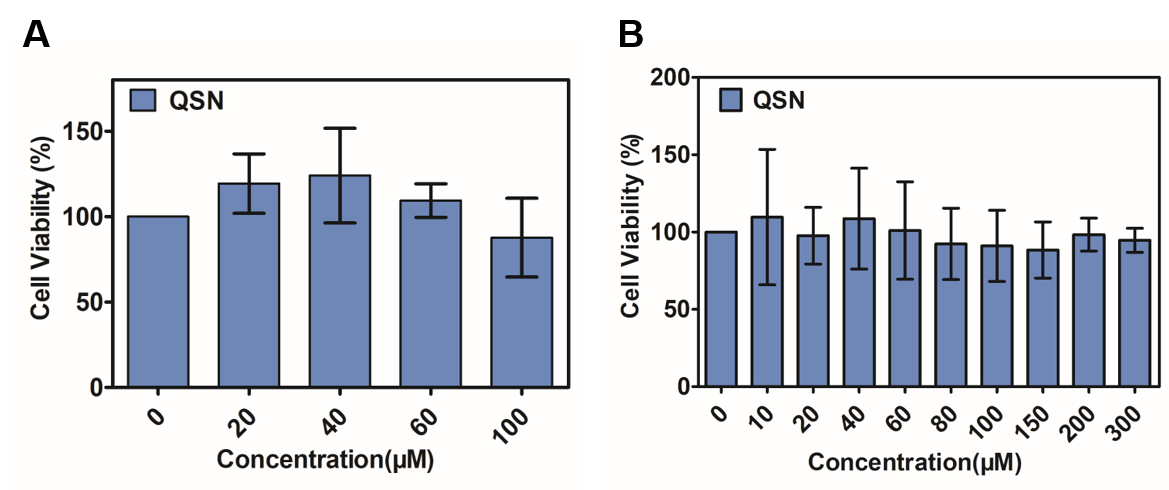


**Figure S4.** A)Cell viability of QSN in H7 at different concentrations after 24 h incubation. Data represent mean values ± standard deviation, n=3. B) Cell viability of QSN in H7NSCs at different concentrations after 15 min incubation. Data represent mean values ± standard deviation, n=3.


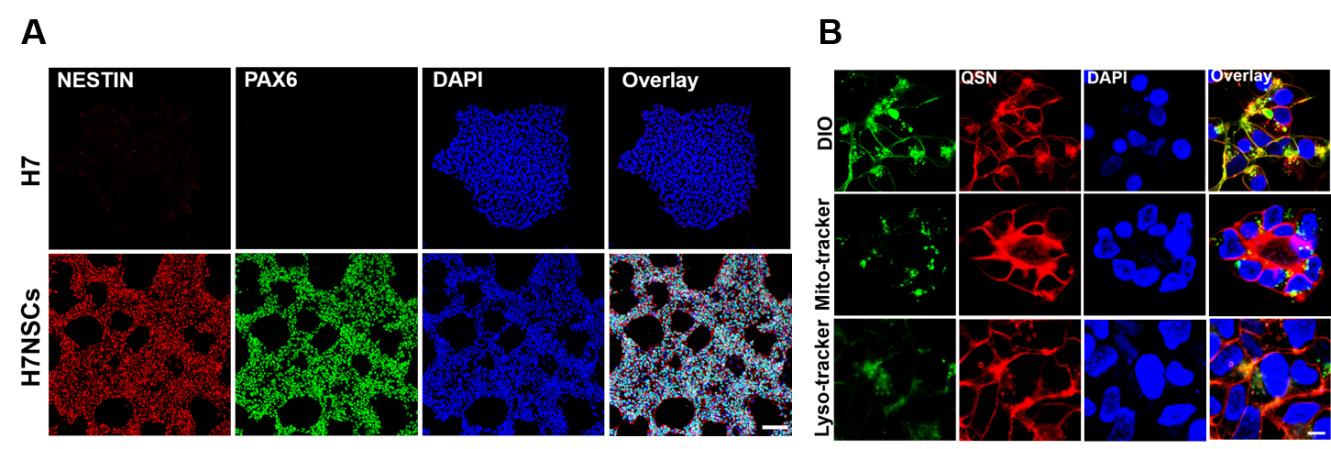


**Figure S5.** A) Differentiation identification for H7NSCs originated from H7. Nestin and pax6 are highly expressed in H7NSCs,while negligible for H7.The scale bar is 100 μm. B) Confocal imaging of H7NSCs co-stained with QSN (20 μM), DIO, Mito tracker, Lysosome tracker. Cells were incubated with QSN for 15 min, and different organelle trackers were added and incubated for additional 15 min to 1 h. For DIO, Mito tracker, they were incubated with cells for 15 min. For Lysosome tracker, it was incubated with cells for 1 h according to the protocols. All scale bars are 5 μm. All experiments were carried out three times independently.


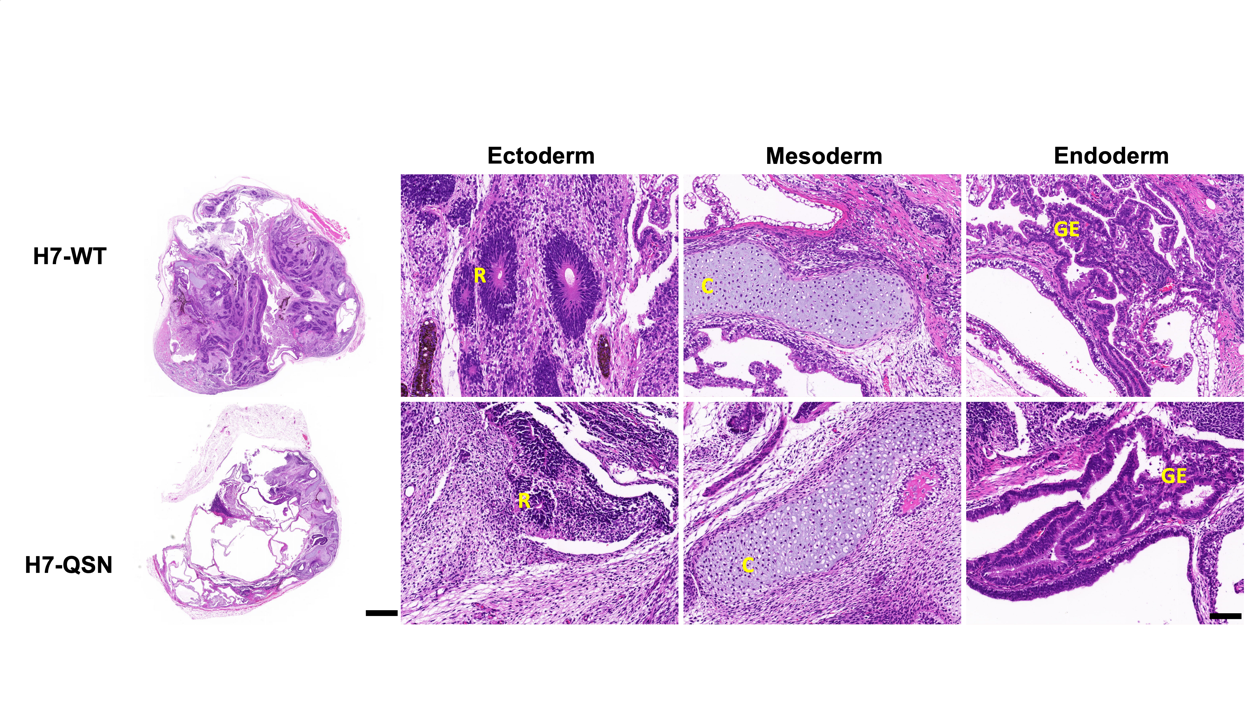


**Figure S6.** Teratoma formation assay. Various cell lineages were present in the teratomas formed by H7-WT and H7-QSN in NCG mice. R, rosette; C, cartilage; GE, gut-like epithelium. The scale bar is 800 μm (left) and 50 μm (right).


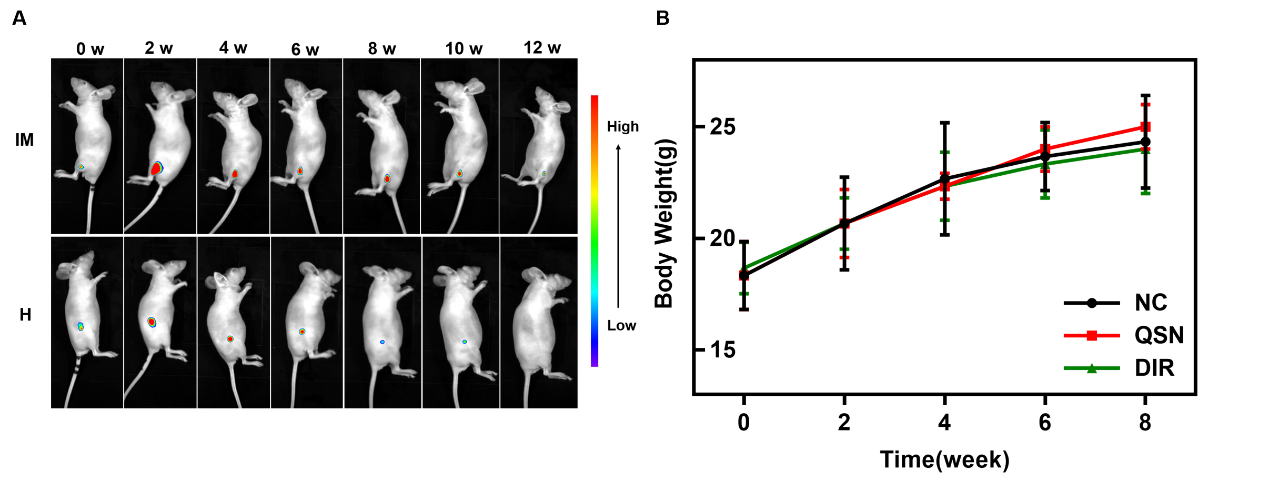


**Figure S7.** A) Long-term in vivo imaging of time-dependent whole-body imaging of H7NSC-bearing nude mice (n=3) after treatment with QSN (300 μM) via by intramuscular injection (IM) and hypodermic injection (H). B) Body weight changes of H7NSC bearing nude mice (n=3) after treatment with QSN and DIR.


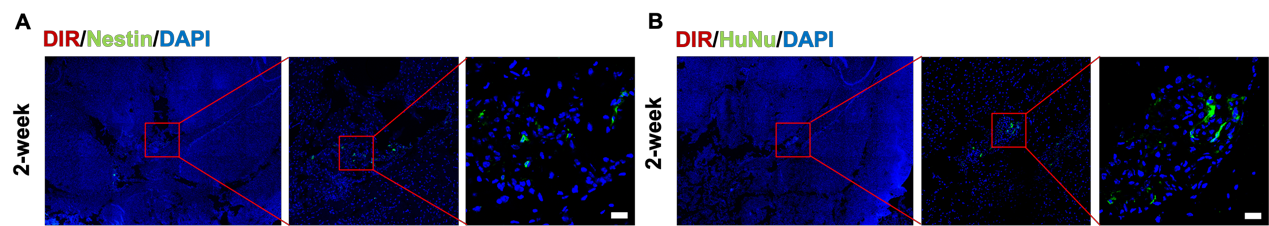


**Figure S8.** Transplantation of DIR-labeled H7NSCs in mouse brain. DIR labeled H7NSCs post-transplantation in mouse brain were stained with A) Nestin , B) human nuclear marker and nuclear marker, DAPI. Scale bar: 20 μm.


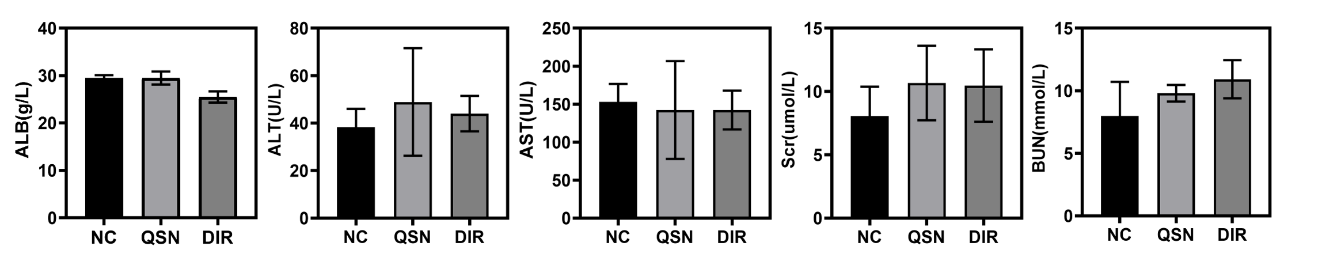


**Figure S9.** Biochemical analysis. Physiological function assessment of hepatic and renal toxicity from normal control, QSN (300 μM) and DIR (1:2500) treatment group. ALB, Albumin; ALT, Alanine transaminase; AST, Aspartate transaminase; Scr, serum creatinine; BUN, Blood urea nitrogen.


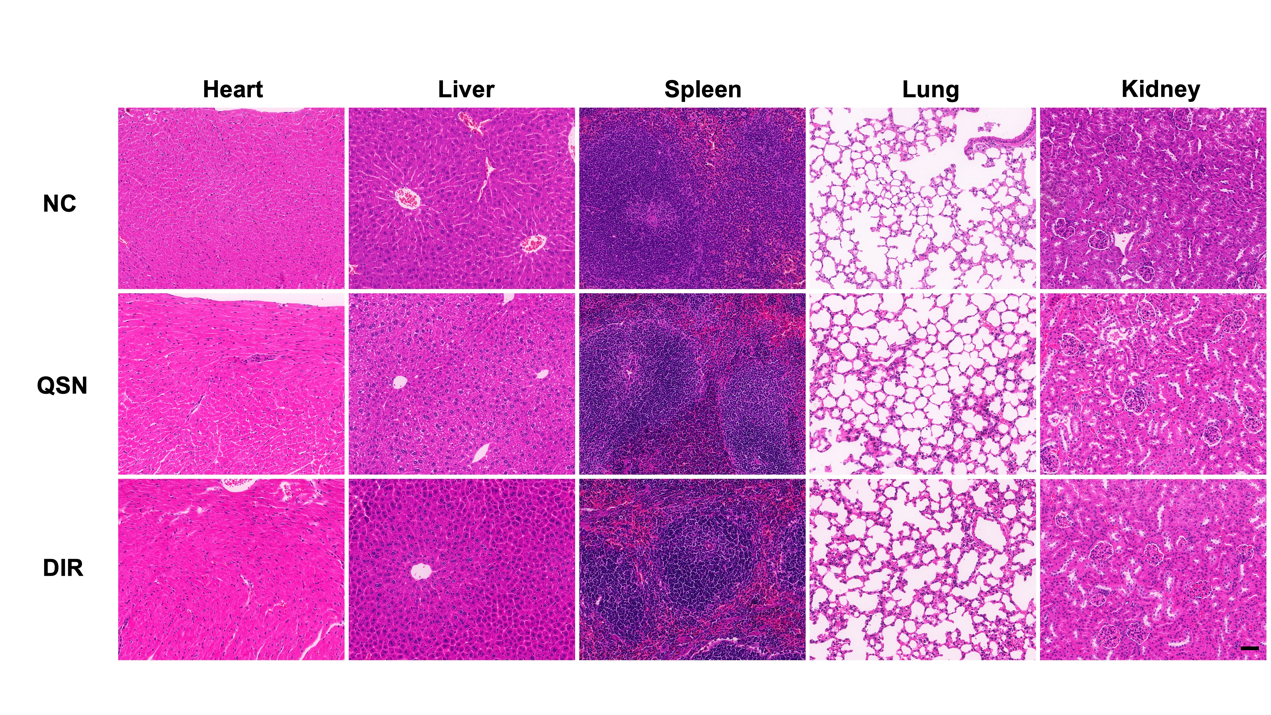


**Figure S10.** Microscopic images of H&E-stained sections of the major organs (heart, liver, spleen, lung, kidney) after nude mice being treated with different material. The scale bar is 50 μm.
